# Two new species of the leafhopper genus *Calodia* Nielson (Hemiptera, Cicadellidae, Coelidiinae) from China, with a checklist and key to Chinese species

**DOI:** 10.3897/zookeys.1023.59811

**Published:** 2021-03-10

**Authors:** Xian-Yi Wang, Zi-Zhong Li, Ren-huai Dai

**Affiliations:** 1 Institute of Entomology, Guizhou University; Guizhou Provincial Key Laboratory for Agricultural Pest Management of the Mountainous Region, Guiyang 550025, China Guizhou University Guiyang China

**Keywords:** Distribution, identification key, morphology, new combination, *
Olidiana
*, taxonomy

## Abstract

Two new species of the leafhopper genus *Calodia* Nielson are described and illustrated: *C. quadrimacula***sp. nov.** from Guizhou and Yunnan Provinces and *C. zuoae***sp. nov.** from Yunnan Province, China. A checklist along with distribution and a key to species based on male genitalia of the genus *Calodia* from China are provided. *Olidiana nigritibiana* (Li), **comb. nov**. (earlier in the genus *Calodia*) is proposed. At present, this genus comprises 45 known species worldwide, of which 19 species are recorded from China.

## Introduction

The genus *Calodia* (Hemiptera, Cicadellidae, Coelidiinae) was described by Nielson (1982) with *Calodia multipectinata* as the type species. *Calodia* is a relatively small genus of leafhoppers widely distributed throughout Asia and also the Pacific (Indonesia and the Philippines). In recent taxonomic studies on Coelidiinae, Nielson (2015) revised the classification of Coelidiini by reassigning the species to several new genera and dealt with six new species of *Calodia*, provided a revised key to species of *Calodia* and also an updated catalogue of the species; of these, two species were from China. Nielson (2015) also resurrected *Lodiana nigritibiana* Li, 1988 and placed it in the genus *Calodia*. Li and Fan (2017) and Viraktamath and Meshram (2019) described new species of the genus from China and India, respectively. So far, there has been a total of 17 species from China.

In this paper, two new species of *Calodia* from China are described together with a checklist to Chinese species of the genus and a key for their separation. *Lodiana nigritibiana* Li, 1988 was resurrected from synonymy and transferred to the genus *Calodia* by Nielson (2015: 13, 83), however, examination of the aedeagus of this species shows that it has one apical process which is bifurcate apically, a character of a number of species of *Olidiana*. Therefore, *Olidiana nigritibiana* (Li), comb. nov. is proposed here.

## Materials and methods

All specimens described in this study were collected by sweep net. Morphological terminology follows mainly Nielson (2015). Other methods follow Fan et al. (2014). Habitus photographs were obtained using a Keyence VHX-6000 system. Illustrations of male genitalia were drawn with Adobe Illustrator CS6 software. These images were combined using the photomerge command in Adobe Photoshop CS6 software.

The type specimens of the new species and other materials examined are deposited in the Institute of Entomology, Guizhou University, Guiyang, China (GUGC), under the following accession numbers: *C. quadrimacula* sp. nov.: #CCW9043; *C. zuoae* sp. nov.: #CCW9065.

## Taxonomy

### Genus Calodia

Taxon classificationAnimaliaHemipteraCicadellidae

Nielson, 1982

56CDBF9F-3A26-55C8-9A30-6481296EC0C0

Calodia Nielson, 1982: 14.

#### Type species.

*Calodia multipectinata* Nielson, 1982.

**Diagnosis.** The species of the genus *Calodia* can be recognized among the genera of Coelidiini by the aedeagus having two medial or apical to subapical processes glabrous or with multi-spinose or multi-setose secondary processes (Nielson 2015).

### Checklist of *Calodia* species from China

***Calodia ailaoshanensis* Li & Fan**

*Calodia ailaoshanensis* Li & Fan, 2017: 37, fig. 7.

Distribution: China (Yunnan).

***Calodia apicalis* Li**

*Calodia apicalis* Li, 1989: 3, figs 20–24; Li and Wang 1991: 114, fig. 58; Zhang 1994: 123; Fan et al. 2014: 98; Nielson 2015: 5; Li and Fan 2017: 39, fig. 8.

Distribution: China (Guizhou).

***Calodia bispinea* Li & Fan**

*Calodia bispinea* Li & Fan, 2017: 41, fig. 9.

Distribution: China (Yunnan).

***Calodia curveprocessa* Li & Fan**

*Calodia curveprocessa* Li & Fan, 2017: 43, fig. 10.

Distribution: China (Yunnan).

***Calodia expenda* Li & Fan**

*Calodia expenda* Li & Fan, 2017: 45, fig. 11.

Distribution: China (Yunnan).

***Calodia forkstyla* Li & Fan**

*Calodia forkstyla* Li & Fan, 2017: 47, fig. 12.

Distribution: China (Yunnan).

***Calodia fusca* (Melichar)**

*Jassus fusca* Melichar, 1903;179.

*Jassus pauperculus* Spangberg, 1878: 35. Synonymised by Nielson (1982).

*Tettigonia frontalis* Kirby, 1891: 171. Synonymised by Nielson (1982).

*Calodia fusca* (Melichar): Nielson 2015: 1982: 156, figs 498–503; Zhang 1994: 123, fig. 121.

Distribution. China.

***Calodia guttivena* (Walker)**

*Coelidia guttivena* Walker, 1857: 99.

*Jassus guttivena* (Walker), Distant 1908: 149.

*Calodia guttivena* (Walker), Nielson 1982: 160, figs 522, 523; Li and Wang 1991: 273; Zhang 1994: 120, fig. 118; Fan et al. 2014: 98; Nielson 2015: 6

Distribution: China (Fujian), Malaysia, Thailand.

***Calodia harpagota* Zhang**

*Calodia harpagota* Zhang, 1994: 125, fig. 124; Fan et al. 2014: 98; Nielson 2015: 5; Li and Fan 2017: 49, fig. 13.

Distribution: China (Shaanxi, Yunnan).

***Calodia lii* Zhang**

*Calodia lii* Zhang, 1994: 123, fig. 120; Fan et al. 2014: 98; Nielson 2015: 5; Li and Fan 2017: 51, fig. 14.

Distribution: China (Tibet).

***Calodia longilamina* (Zhang)**

*Lodiana longilamina* Zhang, 1994: 88 fig. 83.

*Calodia longilamina* (Zhang), Nielson 2015: 7.

Distribution: China (Yunnan).

***Calodia longispina* Li & Wang**

*Calodia longispina* Li & Wang, 1991: 116, fig. 60; Zhang 1994: 123; Fan et al. 2014: 98; Nielson 2015: 5; Li and Fan 2017: 51, fig. 15.

Distribution: China (Guizhou).

***Calodia vincula* Nielson**

*Calodia vincula* Nielson, 2015: 9, 12, Pl. 1C, figs 28 – 32.

Distribution: China (Kouy Tchéou).

***Calodia ostenta* (Distant)**

*Jassus ostentus* Distant, 1918: 49.

*Coelidia ostenta* (Distant), Metcalf 1964: 68.

*Jassus pauperculus* Spangberg, 1878: 35; Ge 1966: 78. Synonymised by Nielson (1982).

Coelidia*paupercula* (Spangberg), Metcalf 1964: 50; Ge 1988: 129.

*Tettigonia frontalis* Kirby, 1891: 169. Synonymised by Nielson (1982).

*Coelidia frontalis* (Kirby), Metcalf 1964: 50.

*Calodia ostenta* (Distant), Nielson 1982: 146, figs 466–469; Li and Wang 1991: 273; Zhang 1994: 119, fig. 117; Fan et al. 2014: 98; Nielson 2015: 6.

Distribution: China (Tibet, Yunnan), India, Sri Lanka.

***Calodia patricia* (Jacobi)**

*Jassus patricius* Jacobi, 1944: 49.

*Coelidia patricia* (Jacobi), Metcalf 1964: 69.

*Jassus ochraceus* Jacobi, 1944: 50. Synonymised by Nielson (1982).

*Coelidia ochracea* (Jacobi), Metcalf 1964: 63.

*Calodia flavinota* Cai & Kuoh, 1993; 219; Nielson 2015: 84. Synonymised by Nielson (2015).

*Calodia paricia* (Jacobi), Nielson 1982: 144; Li and Wang 1991: 273; Zhang 1994: 112, fig. 109; Fan et al. 2014: 97; Nielson 2015: 7. Synonymised by Nielson (1982).

Distribution: China (Fujian, Jiangxi).

***Calodia quadrimacula* sp. nov.**

Distribution: China (Guizhou, Yunnan).

***Calodia scutopunctata* (Zhang)**

*Lodiana scutopunctata* Zhang, 1994: 83, fig. 78.

*Olidiana scutopunctata* (Zhang, 1994) McKamey 2006: 506.

*Calodia scutopunctata* (Zhang, 1994) Nielson 2015:14; Li and Fan 2017: 55, fig. 16.

Distribution: China (Shaanxi, Yunnan).

***Calodia sichuanensis* Nielson**

*Calodia sichuanensis*Nielson 2015: 9, plate 1B, figs 20 – 27.

Distribution. China (Sichuan).

***Calodia zuoae* sp. nov.**

Distribution: China (Yunnan).

### Key to species of *Calodia* (males) from China

**Table:** 

1	Aedeagal shaft with two short processes	**2**
–	Aedeagal shaft with two long processes	**3**
2	Processes of aedeagal shaft bifurcate apically (Fig. 1)	***C. guttivena***
–	Processes of aedeagal shaft not bifurcate apically (Fig. 2)	***C. ostenta***
3	Pygofer side with narrowed at apex and produced posteriorly	**4**
–	Pygofer side not narrowed and produced posteriorly	**5**
4	Pygofer apex strongly sinuate (Fig. 3)	***C. curveprocessa***
–	Pygofer apex not sinuate (Fig. 4)	***C. ailaoshanensis***
5	Subgenital plate with apical process	**6**
–	Subgenital plate without apical process	**7**
6	Subgenital plate (Fig. 5) with one tiny process at apex	***C. patricia***
–	Subgenital plate with one apical and one subapical processes (Fig. 6)	***C. bispinea***
7	Aedeagal shaft processes with secondary spines and arise close to apex (Fig. 8)	**8**
–	Aedeagal shaft with either one or both the processes glabrous (Fig. 13)	**10**
8	Style apophysis more than 5 times as long as basal width and longitudinally rugose (Fig. 7)	***C. apicalis***
–	Style at most 3 times longer than basal width (Fig. 10)	**9**
9	Aedeagal shaft proximal process at most 1½ times as long as distal process, with secondary spines before apex sparse, longer than width of process (Fig. 9)	***C. lii***
–	Aedeagal shaft proximal process twice as long as distal process, with secondary processes before apex dense and shorter than width of process (Fig. 30)	***C. zuoae* sp. nov.**
10	Aedeagal shaft processes glabrous (Li and Fan 2017, plate 17, fig. 7)	***C. longispina***
–	One of the aedeagal shaft processes with secondary spines (Fig. 13)	**11**
11	Aedeagal shaft with dorsal margin before apical group of teeth and base of distal process smooth in lateral view (Zhang 1994, fig. 121)	**12**
–	Aedeagal shaft with dorsal margin before apical group of teeth and base of distal process dentate in lateral view (Figs 13, 22)	**13**
12	Aedeagal shaft processes almost equal in length (Zhang 1994, fig. 121)	***C. fusca***
–	Aedeagal shaft with proximal process more than 3 times as long as distal spine-like process (Nielson 2015, fig. 30)	***C. vincula***
13	Style apophysis either bifid (Fig. 20) or bilobed (Li and Fan 2017, plate 14, fig. 8)	**14**
–	Style apophysis neither bifid nor bilobed (Figs 9, 11, 12)	**16**
14	Style apophysis with bilobed apex (Fig. 20)	**15**
–	Style apophysis deeply bifid (Li and Fan 2017, plate 14, fig. 8)	***C. forkstyla***
15	Style apophysis with a subapical spur (Fig. 20)	***C. quadripunctula* sp. nov.**
–	Style apophysis without subapical spur (Li and Fan 2017, plate 15, fig. 8)	***C. harpagota***
16	Style apophysis tapered towards apex (Figs 9, 11, 12)	**17**
–	Style apophysis not tapered towards apex (Figs 11, 12)	**18**
17	Aedeagal shaft distal process with lateral margin serrate (Zhang 1994, fig. 83)	***C. longilamina***
–	Aedeagal shaft distal process with lateral margin smooth (Nielson 2015, fig. 30)	***C. sichanensis***
18	Style apophysis almost of uniform width throughout (Fig. 11)	***C. scutopunctata***
–	Style apophysis widened near apex (Fig. 12)	***C. expanda***

**Figures 1–13.**
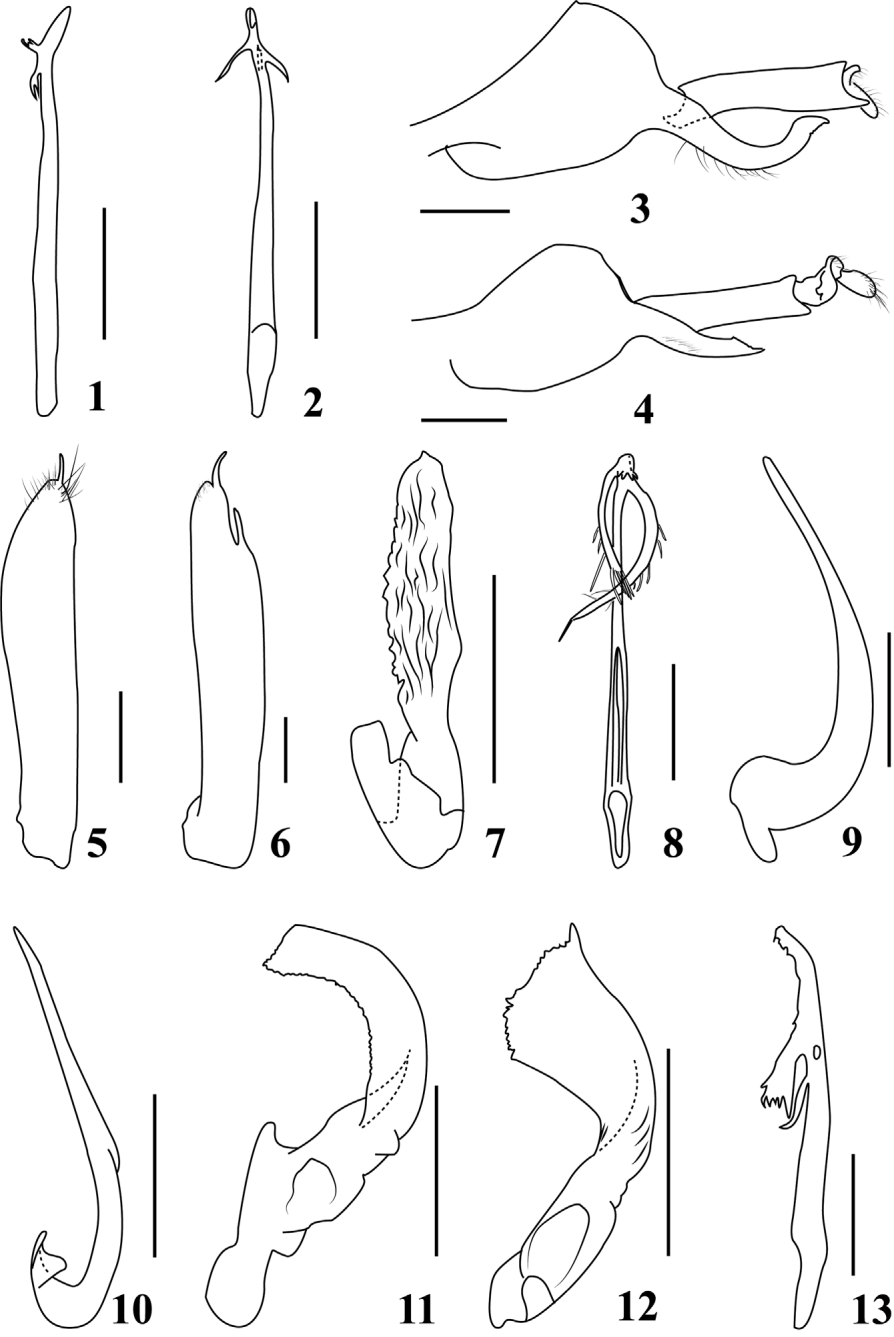
*Calodia* species, male genitalia **1***C. guttivena* (Walker), aedeagus and dorsal connective, dorsal view **2***C. ostenta* (Distant), aedeagus and dorsal connective, dorsal view **3***C. curveprocessa* Li & Fan, pygofer side, lateral view **4***C. ailaoshanensis* Li & Fan, pygofer side, lateral view **5***C. patricia* Jacobi, male subgential plate, ventral view **6***C. bispinea* Li & Fan, male subgential plate, ventral view **7***C. apicalis* Li, style, lateral view **8***C. lii* Zhang, aedeagus and dorsal connective, dorsal view **9***C. longilamina* (Zhang), style, lateral view **10***C. longispina* Li & Wang, style, lateral view **11***C. scutopunctata* (Zhang), style, lateral view **12***C. expenda* Li & Fan, style, lateral view **13***C. harpagota* Zhang, aedeagus and dorsal connective, dorsal view. Scale bars: 0.5 mm.

### Calodia quadrimacula sp. nov.

Taxon classificationAnimaliaHemipteraCicadellidae

F42E4C22-61C5-5BAF-9209-1B0500F35FCD

http://zoobank.org/8C89F5F1-C4DC-4FA5-92ED-6AC3C1D9268F

Figs 14–23

#### Type material.

***Holotype*,** ♂, China: Guizhou Province, Bijie City, Weining County, Caohai Reserve, 3 July 2017, coll. Caohai expedition team (GUGC). ***Paratype***, 3 ♂♂, 6 ♀♀ same information as holotype. 2 ♂♂, CHINA: Yunnan Province, Yuxi City, Xinping County, 21 July 2018, coll. Xian-yi Wang (GUGC).

#### Diagnosis.

The new species is similar to *C. harpagota* Zhang, 1994, but differs in having the style apophysis with a subapical spur and the aedeagal shaft with angular projection on the ventral margin in lateral view and with two slender subapical processes.

#### Description.

Middle-sized species. Body length (including tegmina): male, 7.2–7.8 mm, female, 7.9–8.4 mm.

**Figures 14–23.**
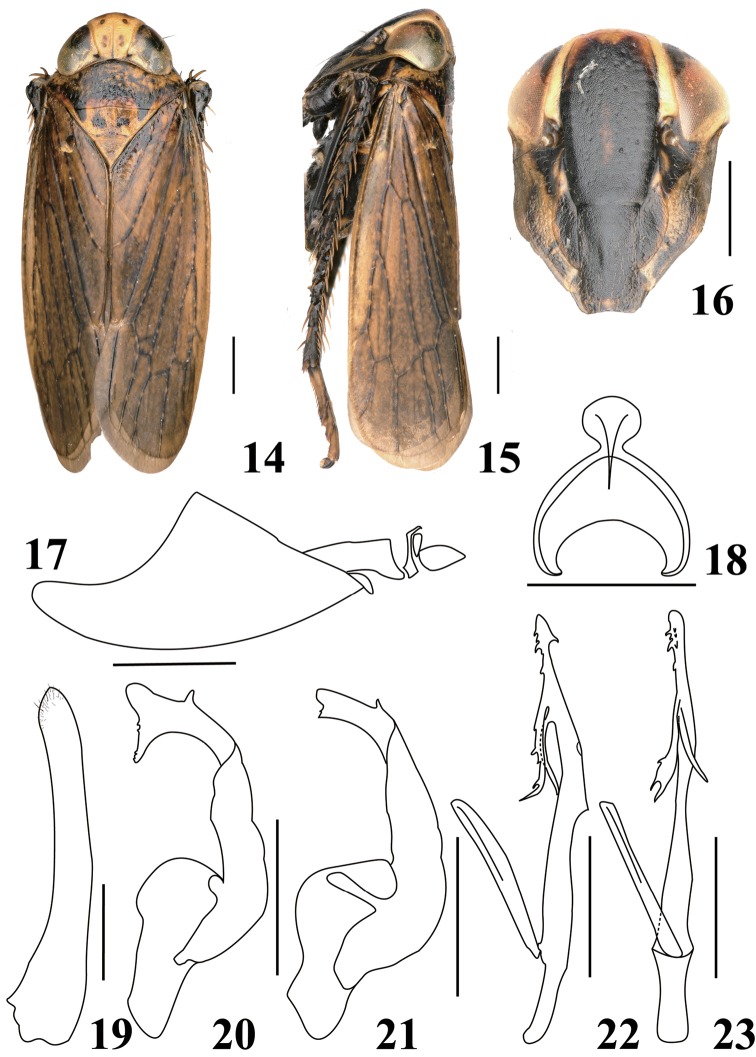
*Calodia quadrimacula* sp. nov. **14** adult, dorsal view **15** adult, lateral view **16** face **17** male pygofer side, lateral view **18** connective, dorsal view **19** male subgential plate, ventral view **20** style, dorsal view (Yunnan) **21** style, dorsal view (Guizhou) **22** aedeagus and dorsal connective, dorsal view **23** aedeagus and dorsal connective, lateral view. Scale bars: 1 mm (**14–16**); 0.5 mm (**17–23**).

***Coloration.*** Ground color brown. Crown yellow with two pairs of brown spots medially, ocelli black (Fig. 14). Face (Fig. 16) with clypeus and clypellus black; area between laterofrontal sutures and eye yellow. Pronotum (Fig. 14) dark brown, with yellow markings. Mesonotum (Fig. 14) with basal triangles and one round spot on either side of median line anterior to scutoscutellar suture, black. Venation black, with numerous, small, brown spots. Legs (Fig. 15) dark brown to black.

***Morphology.*** Head narrower than pronotum, anterior margin broadly obtuse; crown as wide as diameter of one eye, produced anteriorly beyond eyes; ocelli on anterior margin of crown; coronal suture extending to level of ocelli (Fig. 14); clypeus (Fig. 16) flat, narrow, laterally expanded under antennal sockets, apex constricted, base inflated longitudinally, apically with lateral margins expanded. Pronotum (Fig. 14) surface bullate. Mesonotum (Figs 14, 15) nearly as long as pronotum.

***Male genitalia.*** Pygofer with caudal lobe (Fig. 17) broadly triangular in lateral view. Subgenital plate (Fig. 19) long with base slightly broad, sparsely setose apically. Style (Figs 20, 21) well developed, base expanded, apex bilobed, with a subapical short spur. Connective (Fig. 18) Y-shaped with stem short. Aedeagal shaft (Figs 22–23) sinuated, ventral margin in lateral view with angular projection slightly distad of half length, curved apically in lateral view, apex with group of spines, with two subapical retrose processes arising on same side, distal process about twice as long as proximal one, with outer margin, serrate proximal process glabrous; gonopore large, subapical, situated laterally more proximal than proximal process.

#### Etymology.

The new species name is derived from the words “*quadri*” and “*macula*”, referring to the scutellum with four black plaques.

#### Distribution.

China (Guizhou, Yunnan).

### Calodia zuoae sp. nov.

Taxon classificationAnimaliaHemipteraCicadellidae

B9C7A29A-189B-5F21-AB67-84983FA02AC3

http://zoobank.org/681D84A8-5E50-4166-9235-9961CB491DC1

Figs 24–31

#### Type material.

***Holotype*,** ♂, China: Yunnan Province, Lushui County, Pianma town, Mt. Gaoligong, 26 May 2019, coll. Qin Zuo (GUGC). ***Paratype***, ♂, same information as holotype.

#### Diagnosis.

The new species is similar to *C. lii* Zhang, 1994, but differs in the structure of aedeagal shaft processes and the aedeagal shaft.

#### Description.

Moderately large species. Body length (including tegmina): male, 8.8–9.4 mm.

**Figures 24–31.**
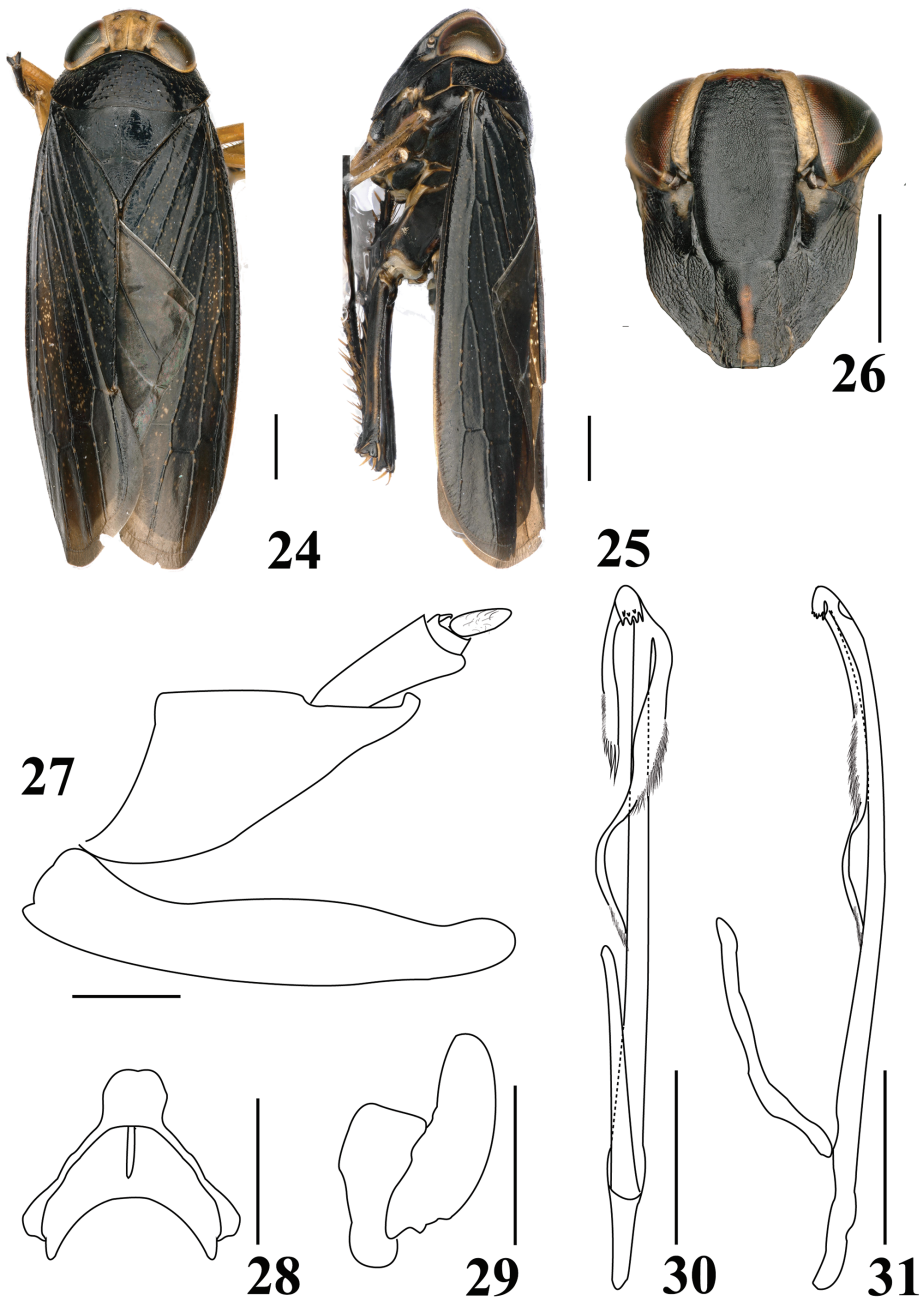
*Calodia zuoae* sp. nov. **24** adult, dorsal view **25** adult, lateral view **26** face **27** male pygofer side and subgential plate, lateral view **28** connective, dorsal view **29** style, dorsal view **30** aedeagus and dorsal connective, dorsal view **31** aedeagus and dorsal connective, lateral view. Scale bars: 1 mm (**24–26**); 0.5 mm (**27–31**).

***Coloration.*** Ground color blackish. Head with crown brown; clypellus with median narrow yellowish stripe; area between lateral frontal sutures and eyes ochraceous (Figs 24, 26). Forewing with numerous, small, ivory to yellow spots.

***Morphology.*** Head, narrower than pronotum, anterior margin broadly rounded; crown broad, slightly broader than width of one eye, slightly produced beyond anterior margin of eyes, eyes about ⅔ width of pronotum (Fig. 24); clypeus wide and short, without middle longitudinal ridge; clypellus slender, apex wider (Fig. 26). Pronotum large, nearly twice as long medially as crown wider than long (Fig. 24). Scutellum large, nearly twice as long medially as pronotum.

***Male genitalia.*** Pygofer in lateral view triangulate, with small lobe apically (Fig. 27). Subgenital plate nearly rectangular, apex rounded (Fig. 27). Style simple, without process (Fig. 29). Connective Y-shaped, stem short (Fig. 28). Aedeagal shaft asymmetrical, slender, narrowly tubular in dorsal view, with two large processes arising almost near apex, shorter process about half as long as longer process, with numerous apical fine setae, longer process extending to midlength of shaft with numerous fine setae on outer margin proximad of midlength; gonopore large, near apex, situated laterally (Figs 30, 31).

#### Etymology.

The new species is named after Ms Qin Zuo who collected the holotype.

#### Remarks.

The new species closely resembles *C. lii* but differs in the structure of aedeagal shaft processes, i.e., aedeagal shaft processes have finer and denser setae in *C. zuoae* compared to sparse and elongate secondary spines in *C. lii*; the shorter process has setae confined to apex of the process in *C. zuoae* and in *C. lii* the spines on the shorter process are along entire lateral margin; the setae on longer process in *C. zuoae* are confined to an area proximad of the midlength on the outer margin of the process and in *C. lii* the sparse spines are found in the distal ¾ length and they are on both margins of the process in the distal ⅓.

## Supplementary Material

XML Treatment for Calodia

XML Treatment for Calodia quadrimacula

XML Treatment for Calodia zuoae
